# A Turkey-origin H9N2 Avian Influenza Virus Shows Low Pathogenicity but Different Within-host Diversity in Experimentally Infected Turkeys, Quail and Ducks

**DOI:** 10.3390/v12030319

**Published:** 2020-03-16

**Authors:** Edyta Świętoń, Karolina Tarasiuk, Monika Olszewska-Tomczyk, Ewelina Iwan, Krzysztof Śmietanka

**Affiliations:** 1 Department of Poultry Diseases, National Veterinary Research Institute, Al. Partyzantów 57, 24-100 Puławy, Poland; karolina.tarasiuk@piwet.pulawy.pl (K.T.); monika.olszewska@piwet.pulawy.pl (M.O.-T.); ksmiet@piwet.pulawy.pl (K.Ś.); 2 Department of Omics Analyses, National Veterinary Research Institute, Al. Partyzantów 57, 24-100 Puławy, Poland; ewelina.iwan@piwet.pulawy.pl

**Keywords:** avian influenza, pathogenicity, diversity, adaptation

## Abstract

Avian influenza virus (AIV) is a highly diverse and widespread poultry pathogen. Its evolution and adaptation may be affected by multiple host and ecological factors, which are still poorly understood. In the present study, a turkey-origin H9N2 AIV was used as a model to investigate the within-host diversity of the virus in turkeys, quail and ducks in conjunction with the clinical course, shedding and seroconversion. Ten birds were inoculated oculonasally with a dose of 10^6^ EID_50_ of the virus and monitored for 14 days. Virus shedding, transmission and seroconversion were evaluated, and swabs collected at selected time-points were characterized in deep sequencing to assess virus diversity. In general, the virus showed low pathogenicity for the examined bird species, but differences in shedding patterns, seroconversion and clinical outcome were noted. The highest heterogeneity of the virus population as measured by the number of single nucleotide polymorphisms and Shannon entropy was found in oropharyngeal swabs from quail, followed by turkeys and ducks. This suggests a strong bottleneck was imposed on the virus during replication in ducks, which can be explained by its poor adaptation and stronger selection pressure in waterfowl. The high within-host virus diversity in quail with high level of respiratory shedding and asymptomatic course of infection may contribute to our understanding of the role of quail as an intermediate host for adaptation of AIV to other species of poultry. In contrast, low virus complexity was observed in cloacal swabs, mainly from turkeys, showing that the within-host diversity may vary between different replication sites. Consequences of these observations on the virus evolution and adaptation require further investigation.

## 1. Introduction

Influenza A viruses show the highest genetic diversity and the broadest host spectrum among the four known influenza virus types [1]. Classification of type A influenza viruses is based on differences in the structure of surface proteins – hemagglutinin (HA) and neuraminidase (NA). A wide range of influenza viruses is found in birds, which can harbour 16 subtypes of HA (H1-H16) and 9 subtypes of NA (N1-N9) in almost all possible configurations [2]. A vast majority of avian influenza viruses (AIV) occur naturally in wild waterfowl in a low pathogenic form (LPAIV) but events of transmission to domestic birds are occasionally reported [3,4,5]. Infections with LPAIV in wild birds are usually asymptomatic [6] but the clinical outcome in poultry varies and is highly dependent on host species. Chickens seem to be most resistant to LPAI infection among the gallinaceous poultry species [7]. Clinical manifestation of LPAI is common in turkeys, and usually involves respiratory system disorders [8,9] often aggravated by secondary bacterial infections [10]. In contrast, infected quail usually shed high amounts of virus in the absence of any clinical signs and mortality, therefore, it is thought that quail may support virus perpetuation and adaptation to other species of gallinaceous poultry [11,12]. 

The high diversity of influenza viruses confers their flexibility, enabling adaptation to novel conditions. The segmented RNA genome may undergo reassortment when two different influenza viruses infect the same cell, potentially generating progeny virions with beneficial gene constellations [13]. Furthermore, the error-prone viral polymerase introduces mutations into the newly synthetized genomes; therefore, the virus exists in a host as a complex swarm of similar but non-identical variants [14]. The heterogeneity and composition of a virus population is a combined effect of mutations generated during replication and selection pressure in the host, but may be also strongly affected by stochastic processes [15,16]. The spectrum of mutants in the virus population determines the adaptability of the virus to changing conditions such as immune response and switching the host species [17]. Therefore, studies on the within- and between-host virus diversity are crucial for the understanding of the virus adaptation, crossing host species barrier, and evolution phenomena [18,19]. This question has been investigated extensively in recent years in the case of influenza infections in humans and other mammals [20,21,22,23,24,25]. However, the within-host diversity of avian influenza viruses in birds is poorly explored, given the wide range of hosts and their manifold roles in the evolution and epidemiology of AIV. To address this question, we performed infection experiments in three common poultry species: turkeys, quail and ducks, using an H9N2 LPAIV of Y439-like lineage. The pathobiological features such as clinical signs, mortality, shedding and antibody levels were characterized in conjunction with the analysis of the virus population diversity in oropharyngeal and cloacal swabs.

## 2. Materials and Methods

### 2.1. Virus Propagation

The H9N2 low pathogenic avian influenza virus A/turkey/Poland/14/2013 used in the study was detected in an outbreak in a commercial turkey farm. Phylogenetic and molecular analysis showed that this strain originated from the wild-bird reservoir but acquired markers of adaptation to gallinaceous poultry [10]. The virus was propagated in 9–11-day-old embryonated specific pathogen free (SPF) chicken eggs. The virus stock was titrated by inoculation of SPF eggs with ten-fold dilutions of virus stock and the 50% embryo infectious dose (EID_50_) was calculated with the Reed and Muench method.

### 2.2. Birds and Infection Experiments 

Experiments were performed in 3-week-old Japanese quail, meat turkeys and Pekin ducks. Turkeys and ducks were purchased in local hatcheries and reared in the animal facility at the National Veterinary Research Institute (NVRI), Poland. Quail were obtained from a commercial farm at the age of two weeks and were housed for one week to allow acclimatization. The experiment for each species was conducted separately in the animal facility of BSL3+ containment level at NVRI. Birds were randomly allocated to three groups: inoculated (10 birds), contact (2 or 3 birds) and control (5 birds), and housed in separate rooms in metal grid cages. Water and feed were provided *ad libitum*. Prior to experimental inoculation, swabs and serum samples were collected and tested by means of real time RT-PCR and ELISA, respectively, in order to exclude any previous AIV infection.

Groups of ten birds were inoculated intranasally and intraocularly with the virus dose of 10^6^ EID_50_ in 0.1 ml divided equally between both infection routes (50 µl split between the two nostrils and 50 µl split between eyes, applied dropwise using a pipette). At 1 day post infection (dpi) three (ducks, turkeys) or two (quail) contact birds of the same species were introduced into the cage with inoculated birds. The control groups consisted of five mock-infected birds of the respective species. Birds were monitored for 14 days for the presence of clinical signs and mortality. At 2, 3, 4, 5, 6, 7, 10 and 14 dpi oropharyngeal and cloacal swabs were collected from all birds using commercial swabs with viral transport medium (Copan, Italy). Additionally, samples of lungs, trachea, duodenum, cecal tonsils and spleen were collected from a turkey that died during the experiment. At 14 dpi blood samples were collected and birds were humanely euthanized.

The animal experiments were approved by the Local Ethical Committee (approval no. 88/2015 of 07 July 2015). 

### 2.3. Evaluation of Virus Shedding and Seroconversion

Extraction of RNA from swab samples was performed with Viral Mini Kit (Syngen, Poland) according to the manufacturer’s manual. The quantity of viral RNA was tested in one-step real time RT-PCR with primers and probe specific for the influenza M gene [26]. Reactions were performed using QuantiTect Probe RT-PCR Kit (Qiagen, Germany) in 7500 Fast Real Time PCR System (Applied Biosystems, USA) with the following protocol: 50 °C for 30 min and 95 °C for 15 min followed by 40 cycles of 95 °C for 10 s, 60 °C for 30 s and 72 °C for 10 s. Ten-fold dilutions of the virus suspension used for inoculation were tested in parallel with the experimental samples to generate the standard curve. The quantity of viral RNA in swabs was expressed as equivalents of EID_50_ (eqEID_50_) in 1 mL of swab medium. Samples of organs collected from the dead turkey were homogenized in PBS to 20% (w/v) suspension and centrifuged (2000 g, 10 min). The RNA was extracted from the supernatant and the quantity of viral RNA in 0.1 g of organ was determined in real time RT-PCR as described above.

Sera collected from birds at 14 dpi were tested with hemaglutination inhibition (HI) method using A/turkey/Poland/14/2013 antigen according to a standard procedure [27]. 

The shedding patterns in inoculated birds were characterized by the following parameters: latency period (time from inoculation to shedding), duration of shedding (number of days in which shedding was identified), peak shedding (maximum amount of virus excreted at any sampling day) and mean daily shedding (mean amount of virus excreted during the shedding period). Since some observations were right censored or interval censored, the following assumptions for the assessment of the duration of shedding were made: if shedding was detected at the last sampling day (14 dpi), this day was designated as the last day of shedding; if shedding stopped between two nonconsecutive sampling days, the last day with positive result was defined as the last day of shedding. The data were generated individually for each bird and shedding route, and mean and standard deviations were calculated. 

### 2.4. Deep Sequencing 

To compare the diversity of viral populations generated in the course of infection in the studied poultry species, swabs collected at 2 and 4 dpi, and the virus stock used for inoculation were subjected to deep sequencing. 

The genome segments were amplified in RT-PCR using SuperScript III One-Step RT-PCR System with Platinum Taq High Fidelity DNA Polymerase (ThermoFisher Scientific) with primers annealing to the conservative 5’ and 3’ termini [28] modified to improve amplification of segments 1–3 [29]. In brief, two reactions were performed for each sample using primers MBTuni-12 (5’-ACGCGTGATCAGCRAAAGCAGG-3’), MBTuni-12G (5’-ACGCGTGATCAGCGAAAGCAGG-3’) and MBTuni-13 (5’-ACGCGTGATCAGTAGAAACAAGG-3’). Primers MBTuni-12 and MBTuni-13 were used in the first reaction, and the second reaction, expected to favour amplification of long segments, contained primers MBTuni-12G and MBTuni-13. The products of both reactions were combined in equal volumes and cleaned with QIAquick PCR Purification Kit (Qiagen) according to the manufacturer’s protocol. The quantity and purity of DNA was measured with NanoDrop 2000 (ThermoFisher Scientific) and Qubit fluorometer (ThermoFisher Scientific). Libraries were prepared using Nextera XT DNA Library Preparation Kit following the manufacturer’s manual and their quality was examined with Fragment Analyzer (Advanced Analytical). Sequencing was performed in MiSeq (Illumina) using MiSeq Reagent Kit v3 (Illumina) with 1% of sequencer throughput per sample. 

### 2.5. Sequencing Data Analysis 

The quality of raw data generated in MiSeq was evaluated with FastQC software (www.bioinformatics.babraham.ac.uk/projects/fastqc/). Adapters and low-quality fragments were removed with Trimmomatic [30] using 4-base sliding window and quality limit of 30. Only reads longer than 50 bases were retained. Filtered reads were mapped to the consensus sequence of the inoculum virus using BWA [31]. Samtools [32] was used to remove PCR duplicates from the alignment and realignment of reads was performed using LoFreq [33]. The output BAM files were processed with LoFreq and VarScan [34] to identify variants in the virus population with reference to the consensus sequence of the inoculum. Only variants identified by both programs, at positions with coverage of at least 500× and frequency above 2% were considered, except for those above 1% that were present at minimum 2% in another sample. The percentage values computed by VarScan were used in further analysis. The number of single-nucleotide polymorphisms (SNPs) was determined in each sample. Additionally, based on the SNPs frequency, the Shannon entropy was calculated for the virus population in each sample as previously described [35]. 

The nucleotide sequences were translated to amino acids for analysis of nonsynonymous substitutions. The H3 numbering scheme for the mature HA protein is used throughout the manuscript.

### 2.6. Statistical Analysis

The differences in the shedding parameters between species were assessed using U-Mann-Whitney test. The same test was used to compare shedding from respiratory tract and cloaca in each species separately. Comparison of the number of polymorphic positions and Shannon entropy values was also performed with the U-Mann-Whitney test. To evaluate the association between the quantity of viral RNA and the virus diversity in a sample, the Pearson correlation coefficient was calculated for eqEID_50_ and entropy values and was tested statistically. The *p*-value of <0.05 was considered statistically significant in all performed analyses, except for comparison of virus shedding between the three species, when the Bonferroni correction was applied due to multiple comparisons (*p*=0.05/2=0.025).

## 3. Results 

### 3.1. Clinical Signs and Mortality

No clinical signs or mortality were noted in quail and ducks. In turkeys, only slight apathy was observed from 2 dpi until the end of the experiment. One turkey died on 9 dpi. Slight duodenal congestion and cecum distension were found during necropsy. Viral RNA was detected in all sampled organs of this specimen, with the highest viral load in duodenum (1 × 10^5^ eqEID_50_/0.1 g), then in lungs (1.58 × 10^3^ eqEID_50_/0.1 g), spleen (6.3 × 10^2^ eqEID_50_/0.1 g), cecal tonsils (63 eqEID_50_/0.1 g) and trachea (25 eqEID_50_/0.1 g).

### 3.2. Virus Shedding

#### 3.2.1. Turkeys

**Figure 1:**
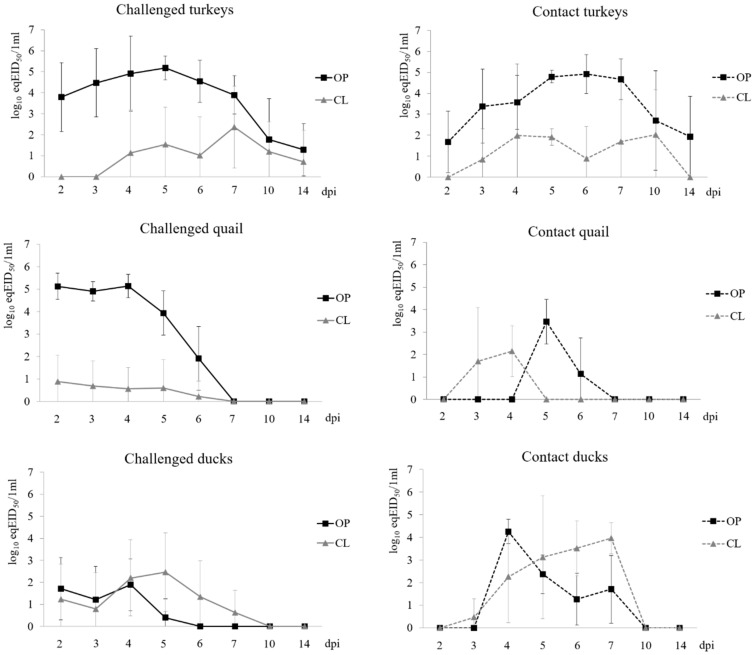
Shedding of H9N2 AIV in challenged (inoculated) and contact (secondarily exposed) turkeys, quail and ducks. OP – oropharyngeal swabs, CL – cloacal swabs.

In the challenged turkeys, shedding from respiratory tract was observed for the whole duration of the experiment (2–14 dpi), with all oropharyngeal swabs positive at 5–7 dpi (Table 1, Figure 1). Shedding from cloaca began later (*p* < 0.001) and was shorter in duration (*p* < 0.01) than oral shedding (Table 1). The overall level (peak and mean) of respiratory shedding was higher than from cloaca (*p* < 0.001). Oral shedding in contact turkeys was observed as soon as at 1 dpc (days post contact) and continued until 13 dpc. Cloacal shedding in contact turkeys was noted at 2–9 dpc.

#### 3.2.2. Quail

Inoculated quail showed oral shedding between 2 and 6 dpi, and at 2–5 dpi viral RNA was detected in all oropharyngeal swabs (Table 1). Cloacal shedding was observed in a total of 6 birds. There was no significant difference in the latency between the oral and cloacal shedding but the former was longer (*p* < 0.01). The amount of virus excreted from respiratory tract was also higher than from cloaca (*p* < 0.01 for peak shedding and *p* < 0.001 for mean shedding). In contact quail, oral and cloacal shedding was demonstrated at 4–5 dpc and 2–3 dpc, respectively.

#### 3.2.3. Ducks

Shedding from respiratory tract was observed in all inoculated ducks and cloacal shedding was found in a total of 8 birds (Table 1, Figure 1). There were no statistically significant differences in terms of the latency, duration and level of oral and cloacal shedding (Table 1). Contact ducks showed oral shedding from 3 dpc and cloacal shedding began at 2 dpc, both continuing until 6 dpc.

#### 3.2.4. Comparison of Shedding in Inoculated Turkeys, Quail and Ducks

No significant differences in the latency of oropharyngeal shedding were observed between the tested species. However, both quail and ducks showed shorter latency period for cloacal shedding than turkeys (*p* < 0.01 and *p* < 0.001, respectively) (Table 1). The highest duration of oral shedding was observed in turkeys and it was significantly longer than in quail (*p* < 0.01) and in ducks (*p* < 0.001). The statistically significant difference for the oral shedding duration was also noted between quail and ducks (*p* < 0.001). It was also demonstrated that cloacal shedding in turkeys was longer than in quail (*p* < 0.025). Comparison of the level of shedding (peak and mean daily shedding) showed no statistically significant differences for turkeys and quail, but both species shed higher amounts of virus via the respiratory route than ducks (*p* < 0.001). However, cloacal shedding in ducks was higher than in quail (*p* < 0.025), but it did not differ significantly from cloacal shedding in turkeys. 

### 3.3. Antibody Response

The HI test revealed seroconversion in all challenged and contact turkeys with titres ranging from 5 log_2_ to 9 log_2_. Positive results of HI test were also found in 7 out of 10 challenged quail and in both contact quail (titres 4–5 log_2_). The lowest response was observed in ducks as five challenged and two contact birds seroconverted (titres 4–5 log_2_).

### 3.4. Diversity of Virus Population in Swabs

In the case of quail, sequencing results were generated for all oropharyngeal swabs from both sampling points. No sequences from cloacal swabs from quail could be obtained due to very low viral load. Sequences of the excreted virus were obtained also for seven (out of nine) and eleven oropharyngeal swabs collected from turkeys at 2 and 4 dpi, respectively. Viruses from three cloacal swabs collected from turkeys at 4 dpi were also sequenced. Samples from ducks, due to generally low viral load, were most problematic in sequencing. Only one sequence from 2 dpi and eight sequences from 4 dpi (3 OP and 5 CL) of sufficient quality were obtained. Additionally, sequencing of virus in cloacal swabs from ducks and turkeys from 5–7 dpi was also performed. For all of the samples tested, a high coverage was obtained for all segments of the viral genome (Supplementary Figure S1).

**Figure 2:**
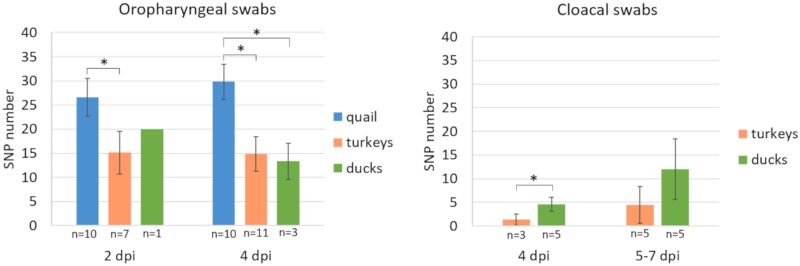
Average numbers of single nucleotide polymorphisms (SNPs) present in virus populations of oropharyngeal and cloacal swabs collected from birds infected with H9N2 AIV. The asterisks (*) show the statistically significant differences.

A total of 22 minority variants (i.e., <50%) were identified in the virus stock used for inoculation. Most of them were located in the HA gene. The highest number of single-nucleotide polymorphisms in the virus population was observed in quail, as 21–32 and 25–37 SNPs distributed across 172 nucleotide positions were identified at 2 and 4 dpi, respectively (Figure 2). These values were significantly higher than the number of SNPs found in oropharyngeal swabs from turkeys at 2 dpi (10–22; *p* < 0.01) and at 4 dpi (9–21; *p* < 0.001). The virus population in the oropharyngeal swab from a duck at 2 dpi contained 20 SNPs and 9–16 SNPs were revealed at 4 dpi, which was significantly lower than in quail at 4 dpi (*p* < 0.01) (Figure 2).

In contrast, virus populations in cloacal swabs collected at 4 dpi showed lower diversity, as a maximum of 2 SNPs and 4–7 SNPs were found in turkeys and ducks, respectively. In both species, these values were significantly lower than in oropharyngeal swabs (*p* < 0.05). To clarify whether this trend was maintained later during the course of infection, additional analysis of cloacal swabs collected between 5 and 7 dpi from turkeys (*n* = 5) and ducks (*n* = 5) was performed. An increase in the number of polymorphic sites was observed, but these were mainly newly emerged variants with low frequency (<5%) (Figure 2).

**Figure 3:**
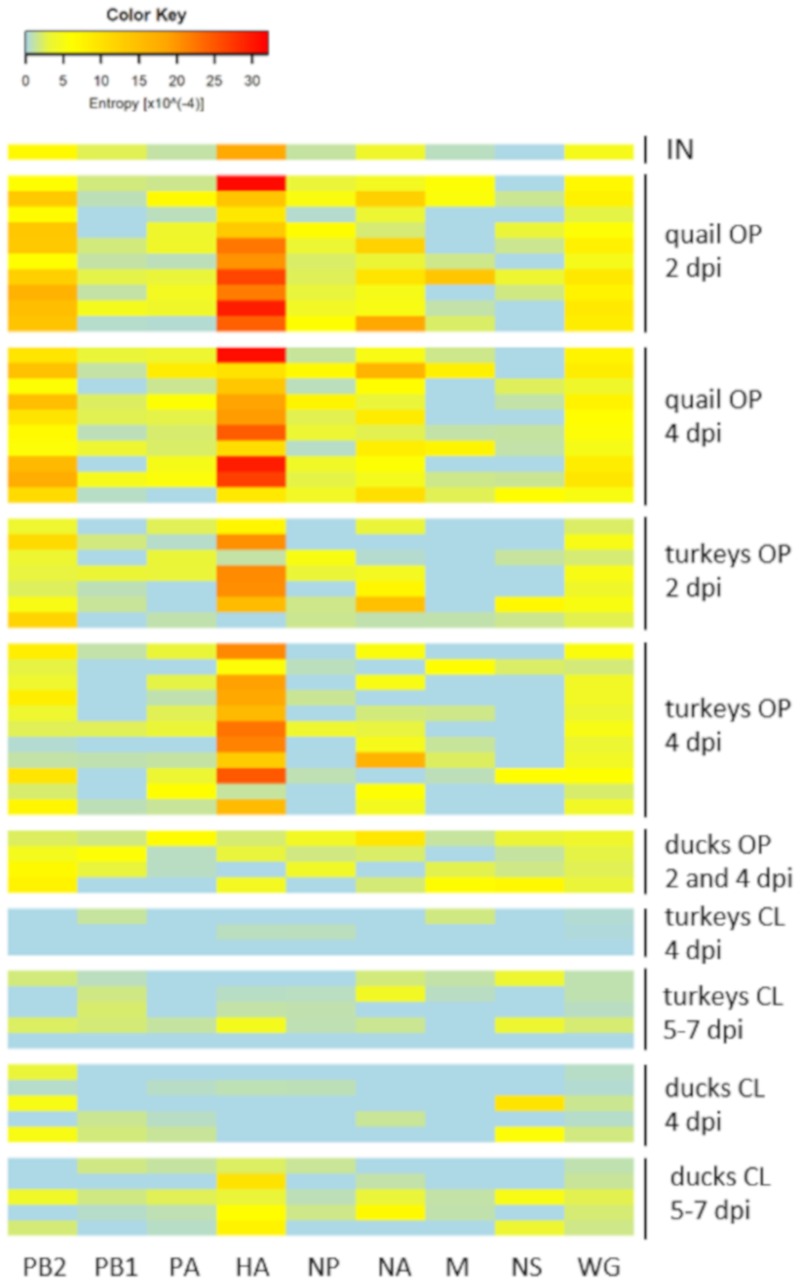
Heatmap of Shannon entropy for each genome segment and the whole viral genome (WG) calculated from the frequencies of variants present in the virus population in oropharyngeal and cloacal swabs collected from turkeys, quail and ducks infected with H9N2 LPAIV. Each row refers to individual sample. IN – inoculum, OP – oropharyngeal swab, CL – cloacal swab.

Based on the frequency of variants in the virus population, the value of Shannon entropy was calculated for each sample and visualized with a heatmap (Figure 3). The results were in agreement with those obtained in the analysis of the SNPs numbers. The highest complexity of virus population was noted in oropharyngeal swabs from quail with significant differences compared to entropy in oropharyngeal swabs from turkeys (*p* < 0.01 at 2 dpi and 4 dpi) and ducks (*p* < 0.01 at 4 dpi). The HA gene contributed mostly to the virus diversity in oropharyngeal swabs, while the lowest complexity was found for the PB1 and NS segments (Figure 3). In contrast, high homogeneity was observed in cloacal swabs collected at 4 dpi, followed by slight increase at 5–7 dpi as a result of the emergence of new low-frequency polymorphisms (Figure 3). The scores for cloacal swabs from 4 dpi were significantly lower than those for oropharyngeal swabs in both turkeys (*p* < 0.01) and ducks (*p* < 0.05).

### 3.5. Synonymous and Nonsynonymous Mutations

Nonsynonymous variants were present in 77 out of 172 polymorphic positions (44.8%) identified in quail, including seven nonsynonymous mutations (PB2-A156V, PB1-K208R, HA-Y17H, HA-I30T, HA-L133R, HA-L133S, HA-T436I) derived from the parental stock. The highest proportion of nonsynonymous substitutions (56.3%) was found in the HA gene. In turkeys the nonsynonymous polymorphisms represented 51.3% (61/119) with seven mutations of inoculum-origin (PB2-A156V, PB1-K208R, HA-Y17H, HA-I30T, HA-L133R, HA-L133S, HA-T436I). The highest percentage of nonsynonymous substitutions was shown in ducks (62.6%; 82/131), wherein two of them were introduced with the inoculum material (HA-Y17H, HA-L133S). 

None of the nonsynonymous mutations found in the HA protein have been linked to the increased affinity to mammalian-type sialic acid receptors.

### 3.6. Changes in the Variant Frequency and Consensus-Level Substitutions

**Figure 4:**
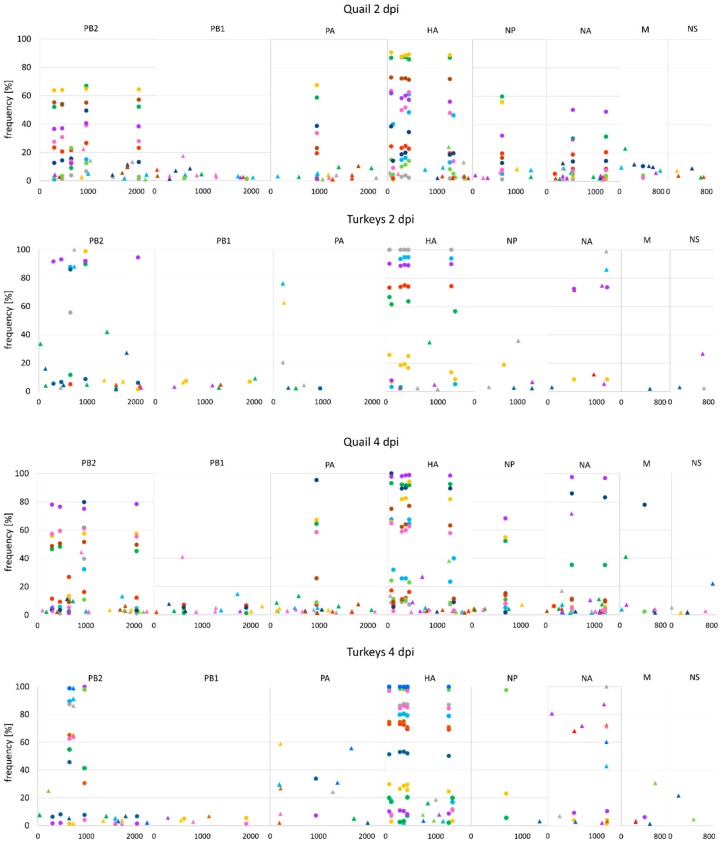
Distribution and frequency of variants in the virus population in oropharyngeal swabs collected from turkeys and quail infected with H9N2 AIV. The x-axis represents the genomic positions for each genome segment. Variants present in the same samples are showed with the same colour. Circles – variants of inoculum origin, triangles – variants that emerged during infection.

The SNPs positions in the original virus stock and in swab samples were compared to differentiate mutations originating from the inoculum from those that arose in birds during infection. Out of 22 minority variants (i.e., <50%) present in the inoculum isolate, 21, 20 and 9 were detected in quail, turkeys and ducks, respectively. A high variation in terms of the frequency of these variants in individual birds was observed (Figure 4).

In quail, changes in the consensus sequences resulted mostly from selection of inoculum-derived mutations, while the vast majority of novel variants were present at low frequencies (<10%) (Figure 4). The consensus-level substitutions (i.e., >50%) were identified in 16 positions, including 4 nonsynonymous mutations, in a total of 7 birds. Except for the mutation in the NA position 534, all these substitutions were present in the inoculum material as minority variants (Table 2, Supplementary Tables). Most of the newly acquired polymorphic sites were found in single birds while the inoculum-origin variants were usually shared by all birds (Figure 4). In contrast, 10 inoculum-independent mutations at the consensus level (4 nonsynonymous) were identified in oropharyngeal swabs from turkeys. Additionally, 16 inoculum-derived substitutions were found, including 6 nonsynonymous, which yielded consensus-level changes at a total of 26 genomic positions (Table 3, Supplementary Tables). In ducks, all low-frequency polymorphisms in the polymerase complex genes, NP, NA and M of the parental isolate were eliminated and only a part of variants in the HA gene was maintained, but they were selected to dominant levels only in two birds. Another two changes in the consensus sequences were noted in PB2 in cloacal swabs from 5–7 dpi (Table 4, Supplementary Tables). The most prominent changes in the dominant subpopulations were observed in cloacal swabs from turkeys. The inoculum variants were either eliminated or increased to a level of 100%. However, there was no noticeable preference for particular variants in the gastrointestinal tract as different patterns were observed in individual birds. The most distinct set of mutations was found in a cloacal swab from a turkey that died at 9 dpi. The complete selection of variants occurred in nine genome positions, including several unique nonsynonymous mutations: E452V in PB2, R187K and K208R in PB1, L133R in HA (Table 3, Supplementary Tables). Interestingly, these substitutions were found also in the oropharyngeal swab from the same bird, but they were present only as low-frequency variants (<5%).

### 3.7. Correlation Between Viral Load and Virus Diversity

No significant correlation was observed between the entropy values and eqEID_50_ in quail (r = 0.23, *p* = 0.3209), turkeys (r = 0.25, *p* = 0.2134) and ducks (r = −0.178, *p* = 0.5421), indicating that the differences in the virus population diversity are independent of the differences in the virus replication level.

## 4. Discussion

Evolution of viruses is a multifactorial process depending on the virus mutation rate, within-host replication dynamics, infection and transmission modes and other epidemiological and ecological factors [36,37,38,39]. These features seem particularly diverse in the case of AIV due to the broad range of susceptible host species and the high virus variability, therefore different AIVs may undergo distinct evolution patterns. For example, it has been shown that avian influenza viruses evolve faster in poultry than in wild birds, which could be partially explained by differences in the ecology of wild birds and poultry resulting in distinct contact rates and transmission routes [40]. The intra-host virus population diversity underlies and affects the between-host virus evolution in a long-term perspective. Therefore, investigations of the within-host virus diversity combined with the data on pathobiology and host ecology may contribute to the understanding of evolution of AIV in this heterogeneous host spectrum. 

In the present study, three common poultry species were infected with an H9N2 AIV isolated from an outbreak in turkeys. Each bird was inoculated with the same virus stock, giving an opportunity to study both the pathogenicity of the virus and how its diversity had been fluctuating during the course of infection. Generally, the pathogenicity of the virus for the studied birds was low, although it varied between the species. Transmission occurred in all tested species as evidenced by shedding and seroconversion in contact birds. The levels of shedding in quail and turkeys were similar to those observed for LPAIV in gallinaceous poultry, i.e., higher amounts shed from respiratory tract than from cloaca [41]. There were no significant differences in the amount of virus shed in turkeys and quail but the former showed longer duration of both oropharyngeal and cloacal shedding. Turkeys were also the only species in which clinical symptoms and mortality were observed. However, the clinical outcome was much milder than in the previous study when infection of turkeys with the same AIV H9N2 strain was exacerbated by concomitant respiratory pathogens [10]. Infections with LPAIV in gallinaceous poultry are usually asymptomatic if uncomplicated, but in turkeys the clinical disease caused by the virus alone is often observed [9,42]. The long duration of respiratory shedding and the robust antibody response indicate that the H9N2 virus may readily infect turkeys and induce only non-specific clinical signs, enabling the spread of the virus within a flock. In addition, it was shown that the virus diversity was sustained during replication in the respiratory tract and a distinct set of novel consensus-level mutations emerged early in the experiment, suggesting a fast evolution of the virus in the course of infection. 

The outcome of H9N2 AIV infection in quail, i.e., the lack of clinical signs with high level of respiratory shedding, is in agreement with the results of other experimental studies on the pathogenicity of LPAIV for this species [11,12]. It was shown that quail are susceptible to infections with most AIV subtypes of wild-bird and mammalian origin [12,43,44]; usually the infectious dose required for efficient replication is lower than for chickens and turkeys [7,11] and shedding from respiratory tract is higher than in chickens [41]. Furthermore, quail may act as an intermediate host for adaptation of LPAIV from wild birds to other species of gallinaceous poultry and mammals [45,46,47,48,49,50]. The subclinical infections with high levels of shedding enabling prolonged circulation of the virus and the presence of both avian- and mammalian-type sialic acid receptors for influenza viruses in quail, are considered as the factors underlying the emergence of viruses with novel properties [11,45,50]. In the present study we showed that, apart from being an asymptomatic reservoir, quail may also support the maintenance and generation of high viral diversity, thus increasing the virus’ adaptive potential. The high complexity of the virus population in oropharyngeal swabs from quail resulted mainly from the preservation and amplification of the original viral diversity, but quail showed also the highest propensity for the generation of novel low-frequency variants. Similar observations were made for Japanese quail infected with clade 2.3.4.4 H5N2 HPAIV as they showed the highest number of polymorphisms in the excreted virus among the tested minor gallinaceous poultry species and chickens [51]. Comparing to chickens and turkeys, the scale of domesticated quail production is small, but it is a common species in mixed backyard holdings, which may support the multipath evolution of the virus once it is introduced into the flock.

In contrast to quail and turkeys, there were no differences in the level of shedding by the oral and cloacal route in ducks, which is in agreement with the results of the meta-analysis performed for various species and LPAIV strain [41]. Furthermore, the high diversity of the inoculum virus was suppressed in ducks. This may have resulted from different selection pressures acting on the virus population in quail and turkeys (representatives of gallinaceous poultry) and genetically distant ducks (representatives of the anseriforms). Similarly, deep sequencing of samples collected from chickens and ferrets infected with a human-origin H7N9 AIV showed high virus heterogeneity in chickens and substantial loss of genetic variation in ferrets, which could be explained by low selection pressure in the natural avian host and strong bottleneck imposed by selection in mammals [18]. A similar effect was observed in chickens and mallards infected with a waterfowl-adapted H5N8 HPAIV, when high diversity of the virus was noted in mallards but lower diversity and higher deviation from the virus inoculum was revealed in chickens [52]. The same mechanism could underlie the severe decline of the inoculum-derived diversity observed in ducks, which were apparently an unfavourable environment for the H9N2 virus which, albeit primordially originating from wild waterfowl, had already undergone adaptation to gallinaceous poultry. A high number of novel variants, mostly nonsynonymous, which emerged in ducks, further supports the hypothesis of strong selection pressure. 

Other interesting findings of the present study were the differences between the variability of virus population in oropharyngeal and cloacal swabs observed mainly in turkeys. The dominant population in cloacal swabs varied between individual birds, indicating that the low diversity could not be attributed to selection of specific variants conferring the best fitness in the gastrointestinal tract. However, a possible explanation of this phenomenon may lie in a “founder effect”. In turkeys, the oropharyngeal shedding preceded the cloacal shedding, as shown by different latency periods, thus it could be expected that the virus population replicating in the intestines was founded by a small proportion of the population present in the respiratory tract, resulting in lower virus diversity. This observation is consistent with population genetics studies, as a spatial spread of a population results in a loss of genetic diversity, and similar effect could be also noticed in the case of dissemination of a virus within a single host [53]. Distinct patterns of AIV diversity in the two replication sites may potentially have consequences on the between-host diversity depending on the transmission route, e.g., in a situation of introduction of a virus with the aerosol route and a further spread of the virus in a flock with a faecal-oral route. Due to the oculonasal infection, the virus reached the respiratory tract first, therefore it would be highly desirable to perform a similar study with a different inoculation route, e.g., oral inoculation in the case of ducks. The experimental setup of the present study did not allow comparison of the diversity between the inoculated and contact birds due to the scarcity of sequences from the contact animals. However, qualitative examination of data generated for two contact turkeys and two contact ducks showed similar diversity in terms of the SNPs numbers and entropy values as those in inoculated birds.

The differences in the within-host virus diversity between the studied species could result from the characteristics of the virus strain used, high diversity of the inoculum stock, infection dose and inoculation route. Since no correlation was found between the virus load and virus diversity, it can be assumed that differences in virus complexity between species or sample types were not caused by differences in the replication level. 

The limited number of studies on the diversity of AIV at the level of individual birds precludes drawing any far-reaching conclusions. Due to the differences in the experimental setup (e.g., infection with HPAI viruses [51,52,54,55]) and the methodology (e.g., data on the virus diversity obtained with deep sequencing [18,51,52], sequencing of amplicons cloned into plasmids [54] or analysis of double peaks in chromatograms [55]), results of these studies cannot be directly compared. However, we show that the within-host diversity of AIV varies between species and noticeable differences may be found even in the same host in various replication sites. Although no detailed analysis of selection pressures was performed in the study, the differences in the proportion of synonymous and nonsynonymous variants and in the number of consensus-level substitutions suggest that varying selection patterns were imposed on the virus population in each species. Further studies are necessary to assess the effect of these discrepancies on the between-host virus evolution and adaptation.

## Figures and Tables

**Table 1:** Shedding patterns in turkeys, quail and ducks infected with A/turkey/Poland/14/2013(H9N2). The number of swabs positive for viral RNA over the total number of swabs tested is presented. OP – oropharyngeal swabs, CL – cloacal swabs, SD – standard deviation.

dpi/dpc	Turkeys				Quail				Ducks			
	Inoculated		Contact		Inoculated		Contact		Inoculated		Contact	
	OP	CL	OP	CL	OP	CL	OP	CL	OP	CL	OP	CL
2/1	9/10	0/10	2/3	0/3	10/10	4/10	0/2	0/2	7/10	5/10	0/3	0/3
3/2	9/10	0/10	3/3	1/3	10/10	3/10	0/2	1/2	5/10	3/10	0/3	1/3
4/3	9/10	3/10	3/3	1/3	10/10	3/10	0/2	2/2	8/10	7/10	3/3	2/3
5/4	10/10	7/10	3/3	3/3	10/10	2/10	2/2	0/2	2/10	7/10	3/3	2/3
6/5	10/10	3/10	3/3	1/3	7/10	1/10	1/2	0/2	0/10	5/10	2/3	3/3
7/6	10/10	7/10	3/3	1/3	0/10	0/10	0/2	0/2	0/10	3/10	2/3	3/3
10/9	5/9	4/9	2/3	2/3	0/10	0/10	0/2	0/2	0/10	0/10	0/3	0/3
14/13	5/9	2/9	2/3	0/3	0/10	0/10	0/2	0/2	0/10	0/10	0/3	0/3
Total	10/10	10/10	3/3	3/3	10/10	6/10	2/2	2/2	10/10	8/10	3/3	3/3
Latency period [days (SD)]	2.3 (0.9)	5.3 (1.3)			2.0 (0.0)	2.5 (0.8)			2.5 (0.8)	2.6 (0.9)		
Duration of shedding [days (SD)]	10.3 (4.2)	4.2 (3.3)			4.7 (0.5)	1.3 (1.6)			2.2 (0.9)	3.0 (2.0)		
Peak shedding [log_10_ eqEID_50_/1mL (SD)]	5.5 (0.5)	3.0 (1.7)			5.4 (0.4)	1.5 (1.4)			2.4 (0.9)	3.4 (1.9)		
Mean daily shedding [log_10_ eqEID_50_/1mL (SD)]	4.4 (0.6)	2.5 (1.3)			4.4 (0.5)	1.3 (1.1)			2.2 (0.8)	2.4 (1.3)		

**Table 2:** Changes in the consensus sequences in samples collected from quail infected with A/turkey/Poland/14/2013(H9N2).

Quail – Oropharyngeal Swabs			
Gene	Position	Amino acid Change	No. of Birds with Frequency >50% at Any Day
PB2	303 ^†^	-	5
	467 ^†^	A156V	5
	966 ^†^	-	6
	2052 ^†^	-	5
PA	948 ^†^	-	4
HA	73 ^†^	Y17H	7
	285 ^†^	-	6
	363 ^†^	-	6
	433 ^†^	L133R L133S	7
	434 ^†^		7
	1260 ^†^	-	6
NP	687 ^†^	-	3
NA	534	-	1
	540 ^†^	-	2
	1218 ^†^	-	2
M	486 ^†^	-	1
^†^ SNPs of inoculum origin			

**Table 3:** Changes in the consensus sequences in samples collected from turkeys infected with A/turkey/Poland/14/2013(H9N2).

Turkeys				
Gene	Position	Amino Acid Change	No. of Birds with Frequency >50% at Any Day	
			Oropharyngeal Swabs	Cloacal Swabs
PB2	303^†^	-	1	-
	467^†^	A156V	1	-
	654^†^	-	6	3
	735	-	5	2
	966^†^	-	4	2
	1355	E452V	-	1
	1740	-	-	1
	2052^†^	-	1	-
PB1	560	R187K	-	1
	623^†^	K208R	-	1
	1911^†^	-	-	1
PA	192	-	1	-
	215	L72S	1	-
	1680	-	1	-
HA	73^†^	Y17H	10	4
	113^†^	I30T	1	1
	285^†^	-	9	3
	363^†^	-	9	3
	433^†^	L133S	9	3
	434^†^	L133R	10	4
	1260^†^	-	9	3
	1334^†^	T436I	1	1
NP	687^†^	-	1	1
	1396	L466I	-	1
NA	84	-	1	-
	540^†^	-	1	1
	544	G182S	1	-
	702	-	1	-
	1114	V372I	1	-
	1156	V386I	1	-
	1203	-	5	2
	1218^†^	-	1	1
M	699	-	-	1
^†^ SNPs of inoculum origin				

**Table 4:** Changes in the consensus sequences in samples collected from ducks infected with A/turkey/Poland/14/2013(H9N2).

Ducks				
Gene	Position	Amino Acid Change	No. of Birds with Frequency >50% at Any Day	
			Oropharyngeal Swabs	Cloacal Swabs
PB2	60	-	-	1
	1139	R380K	-	1
HA	73^†^	Y17H	1	2
	285^†^	-	1	2
	363^†^	-	1	2
	433^†^	L133S	1	2
	434^†^		1	2
	1260^†^	-	1	2
^†^ SNPs of inoculum origin				

## Supplementary Materials

The following are available online at https://www.mdpi.com/1999-4915/12/3/319/s1.
