# Supplementary figures and images for: A Turkey-origin H9N2 Avian Influenza Virus Shows Low Pathogenicity but Different Within-host Diversity in Experimentally Infected Turkeys, Quail and Ducks
Source: Viruses. 2020 Mar 16;12(3):319. doi: 10.3390/v12030319 (PMC7150878; doi:10.3390/v12030319)

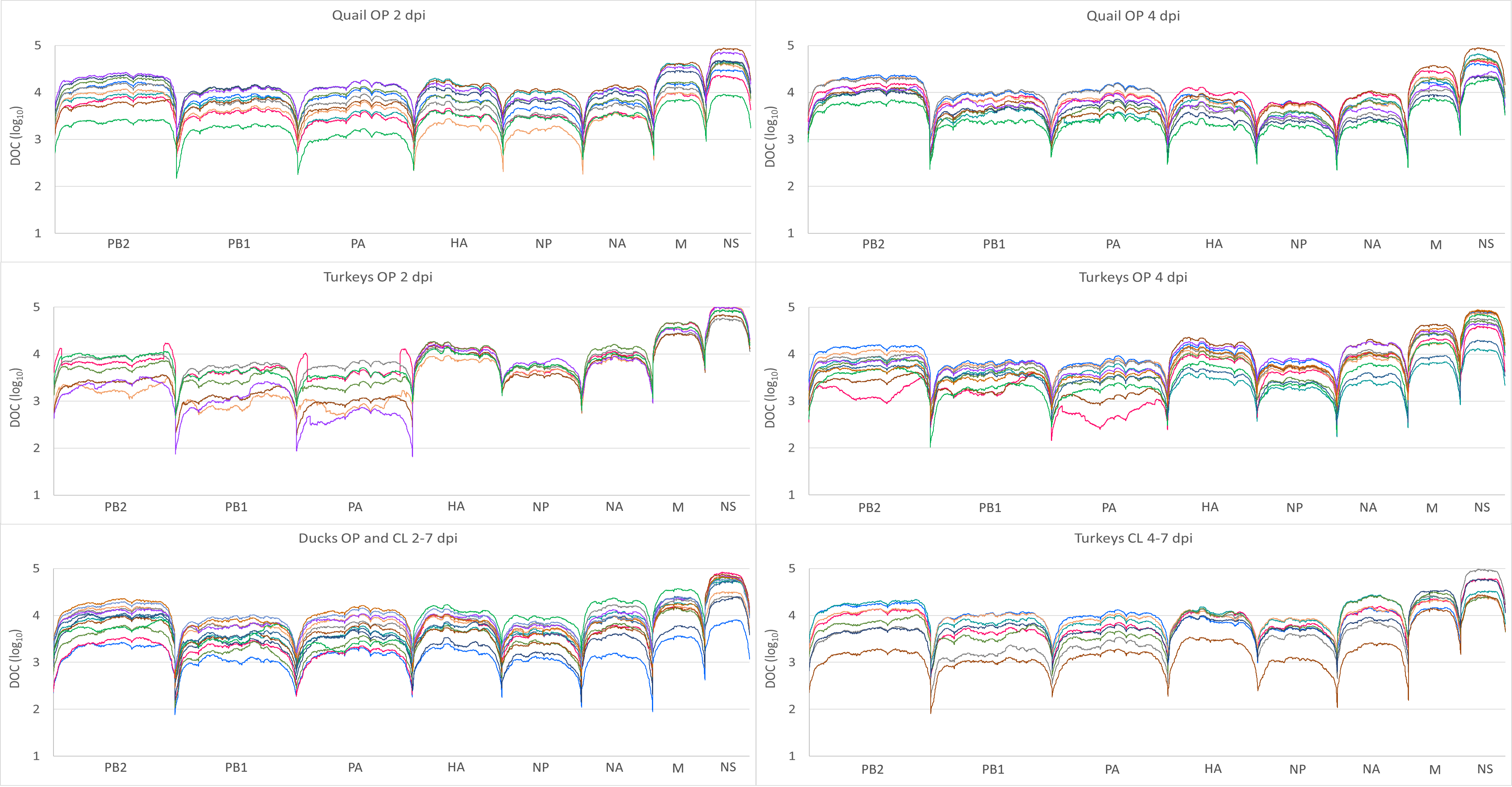

Supplement: Supplementary file 1 [file viruses-12-00319-s001.zip › viruses-747251-supplementary/Figure S1.png]
